# Transcriptome Based System Biology Exploration Reveals Homogeneous Tumorigenicity of Alimentary Tract Malignancy

**DOI:** 10.3389/fonc.2020.580276

**Published:** 2021-01-22

**Authors:** Yu-Chen Lu, Jing-Qi Shi, Zi-Xin Zhang, Jia-Yi Zhou, Hai-Kun Zhou, Yuan-Cai Feng, Zhen-Hua Lu, Shu-Ya Yang, Xi-Yang Zhang, Yang Liu, Zi-Chao Li, Yuan-Jie Sun, Lian-He Zheng, Dong-Bo Jiang, Kun Yang

**Affiliations:** ^1^ Department of Immunology, School of Basic Medicine, The Fourth Military Medical University, Xi’an, China; ^2^ Aviation Psychology Research Office, Air Force Medical Center, Beijing, China; ^3^ Department of Orthopedics, The Tangdu Hospital, The Fourth Military Medical University, Xi’an, China

**Keywords:** alimentary tract malignancy, homogeneous tumorigenicity, transcriptome, competing endogenous RNA, weighted gene co-expression network analysis

## Abstract

Malignancies of alimentary tract include esophageal carcinoma (ESCA), stomach adenocarcinoma (STAD), colon adenocarcinoma (COAD), and rectum adenocarcinoma (READ). Despite of their similarities in cancer development and progression, there are numerous researches concentrating on single tumor but relatively little on their common mechanisms. Our study explored the transcriptomic data of digestive tract cancers from The Cancer Genome Atlas database, yielding their common differentially expressed genes including 1,700 mRNAs, 29 miRNAs, and 362 long non-coding RNAs (lncRNAs). There were 12 mRNAs, 5 miRNAs, and 16 lncRNAs in the core competitive endogenous RNAs network by RNA-RNA interactions, highlighting the prognostic nodes of SERPINE1, hsa-mir-145, and SNHG1. In addition, the weighted gene co-expression network analysis (WGCNA) illustrated 20 gene modules associated with clinical traits. By taking intersections of modules related to the same trait, we got 67 common genes shared by ESCA and READ and screened 5 hub genes, including ADCY6, CXCL3, NPBWR1, TAS2R38, and PTGDR2. In conclusion, the present study found that SERPINE1/has-mir-145/SNHG1 axis acted as promising targets and the hub genes reasoned the similarity between ESCA and READ, which revealed the homogeneous tumorigenicity of digestive tract cancers at the transcriptome level and led to further comprehension and therapeutics for digestive tract cancers.

## Introduction

The incidence and mortality of alimentary tract malignancies have been high in the world, seriously endangering public health and human life (1). For instance, the principle malignant conditions of the digestive tract cause great damage, namely cancers of stomach (approximately 1.0 million new cases in 2018), esophagus (570,000 cases) and colorectum (1.8 million cases) (2). The alimentary tract, from oropharynx to anal canal, are closely related in mainly organ functions and development. No wonder that they should share similar mechanisms in the progression of cancer. However, the mechanisms of digestive tract tumorigenicity in common remain to be explored.

An important reasons why digestive tract cancers contribute to numerous deaths is the lack of suitable and effective diagnosis (2). Besides, for the treatment, the novel strategy such as immunotherapy depends on predictive biomarkers and therapeutic targets pursuing (3). Recently, some molecules closely related to survival rates are considered as potential diagnostic and prognostic tools in digestive tract cancers (4). However, they are not suitable for diagnostic and prognostic biomarkers because of their low specificity and veracity. Thus, it is urged to investigate the molecular mechanisms, which are important for early diagnosis and treatment of digestive tract cancers.

With the development of high-throughput sequencing and omics profiling in life sciences, there are increasing reliable methods and constantly updated databases (5, 6). The Cancer Genome Atlas (TCGA), one of community resource databases, leads frontiers of information to identify novel biomarkers, which has been widely used in cancer research (7, 8). Despite the relative maturity of genomic analyses, researchers believe that progress will primarily come from transcriptome-based subtyping efforts (9). Given that integrated analyses usually emphasis cancer heterogenicity, this study tried another view of homogeneity of cancer development. By using bioinformatics analysis including differentially expressed genes calling, the competitive endogenous RNA (ceRNA) network constructing and the weighted gene co-expression network analysis (WGCNA) exploring, we identified novel biomarkers associated with clinical traits and common regulatory mechanisms of digestive tract cancers.

## Method and Materials

### Download and Pretreat the Data

The RNA-seq and miRNA-seq data were downloaded from the TCGA database using the GDC Data Portal (https://gdc-portal.nci.nih.gov/). The mRNA expression data of ESCA included a total of 171 tumor samples consisting of 160 tumor samples and 11 normal samples. The mRNA expression data of STAD included a total of 407 samples consisting of 375 tumor samples and 32 normal samples. The mRNA expression data of COAD included a total of 177 tumor samples consisting of 167 tumor samples and 10 normal samples. The mRNA expression data of READ included a total of 521 samples consisting of 480 tumor samples and 41 normal samples. The miRNA expression data of ESCA included a total of 198 samples consisting of 185 tumor samples and 13 normal esophageal samples. The miRNA expression data of STAD included a total of 491 samples consisting of 446 tumor samples and 45 normal samples. The miRNA expression data of COAD included a total of 458 tumor samples consisting of 450 tumor samples and 8 normal samples. The miRNA expression data of READ included a total of 84 samples consisting of 83 tumor samples and 1 normal sample. The sequencing data were all publicly available and no ethical issues were involved. The Gencode database (https://www.gencodegenes.org/human/) was used to distinguish and annotate the mRNA and lncRNA from the RNA-seq. LncRNA and mRNA were extracted by Perl to make lncRNA and mRNA data matrixes. The edgeR package in Bioconductor was used to screen the differentially expressed miRNAs, differentially expressed lncRNAs and differentially expressed mRNAs (10). The miRNAs, lncRNA and mRNA were deemed to be different if |log2FoldChange| >0.69 and respectively, both with p-value <0.05. The intersection of four tumors was taken and Venn diagram was drawn to make the common differentially expressed mRNAs, differentially expressed lncRNAs and differentially expressed miRNAs among four tumors.

### Gene Set Enrichment Analysis (GSEA) of Different Cancers

For the differentially expressed mRNAs of four digestive tract cancers, Gene set enrichment analysis (GSEA) was conducted to investigate the hallmarks of the four digestive tract cancers by online tool WebGestalt (http://www.webgestalt.org/). The hallmarks gene sets were downloaded from Molecular Signature database (https://www.gsea-msigdb.org/gsea/msigdb). And p-value <0.05 and FDR <0.05 were set as the cutoff criterion to screen the prominent hallmarks.

### Functional Annotation

We performed GO and KEGG pathways enrichment analysis to detect the potential biological functions and pathways of the common differentially expressed mRNAs using DAVID (https://david.ncifcrf.gov/) online tool (11, 12). And p-value <0.0001 was set as the cutoff criterion. The cytoscape BiNGo plug-in was also used to analysis and draw the results of enrichment (13).

### The Construction of Competing Endogenous RNA (ceRNA) Network

We used miRWalk 3.0 database (http://mirwalk.umm.uni-heidelberg.de/), including Targetscan database, miRDB database and miRTarBase database, aiming to predict target mRNA of different expressed miRNA and took the intersection between the predicted mRNA and common differentially expressed mRNA. We got the adjusted relationships between the miRNA and mRNA. We used the miRcode database (http://www.mircode.org/) to obtain the relationships of lncRNA-miRNA and took the intersection between the predicted lncRNA and common differentially expressed lncRNA. Thus, the visual ceRNA network was constructed by cytoscape software (14). According to ceRNA hypothesis, miRNA expression was negatively correlated with lncRNA or mRNA (15). Thus, for identification of potential ceRNA regulatory axes, the positively correlated lncRNA-miRNA pairs, and miRNA-mRNA pairs in the ceRNA network were discarded. The R package ggalluvial was used to demonstrate the ceRNA axes.

To discover the prognostic factors, we performed the overall survival of genes in the ceRNA network. After downloading the clinical data from TCGA, the R packages survival was used for survival analysis. To avoid the effect of different cancers, we put the tumor samples which expressions were first quarter of each cancer together, treating as the high-expression group. Thus, the hsa-mir-145 high-expression group includes 106 COAD samples, 36 ESCA samples, 38 READ samples, and 100 STAD samples. Besides, the SERPINE1 and SNHG1 high-expression group includes 110 COAD samples, 36 ESCA samples, 39 READ samples, and 94 STAD samples. Just like the high-expression group, we set the low-expression group with the same numbers of samples. The Log-rank test was used to overall survival analysis and we selected the genes with P value <0.05.

For the lncRNA of highest connectivity, the GEPIA2 database (http://gepia2.cancer-pku.cn/#index) was used to show co-expression relationships between lncRNA and mRNA. Besides, the ImmLnc database (http://bio-bigdata.hrbmu.edu.cn/ImmLnc/) was used to show the Immune-related pathways.

### The Weighted Gene Co-Expression Network Analysis (WGCNA)

The “WGCNA” package in R software was used to construct gene co-expression network and identify the co-expression genes modules (16). For four tumors, all genes were divided into different modules (colors) through cluster analysis and dynamic tree cut algorithm. We combined the gene matrixes with clinical traits to analyze the correlation between the different genes modules and clinical traits. We set the |correlation score| >0.3 and P <0.05 as threshold values. For the different gene modules of different cancers, which were related to same clinical trait, we took the intersection to get the common genes. For the common genes, STRING database (https://string-db.org/) was used to construct the PPI network and cytoscape MCODE plug-in was used to screen the hub genes (17). And Metascape database (https://metascape.org/) and David database (https://david.ncifcrf.gov/) were used to perform the GO and KEGG pathways enrichment analysis to detect the potential biological functions of common genes (11, 12). For the hub genes, the UALCAN database (http://ualcan.path.uab.edu/) was used to analyze the correlation between hub genes expression and clinical traits. For the methylation analysis, We explored the association between hub genes’ expression levels and their methylation status by Disease Meth version 2.0 (http://bio-bigdata.hrbmu.edu.cn/diseasemeth/) and MethSurv (https://biit.cs.ut.ee/methsurv/). Besides, the online database TIMER2.0 (http://timer.cistrome.org/) was used to verify that the above key nodes in ceRNA network and hub genes were the common features in alimentary tract malignancies.

## Result

### Differentially Expressed Genes in ESCA, STAD, COAD, and READ Based on TCGA Data

**Figure 1:**
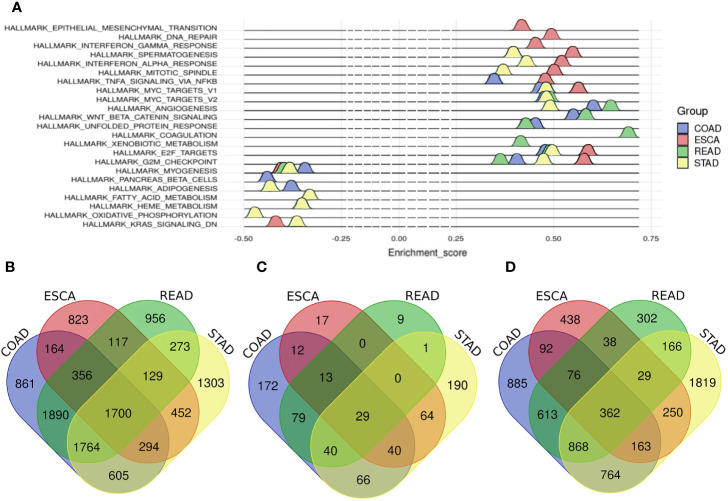
The hallmarks of different tumors and Venn diagram of common m RNAs, common miRNAs and common lncRNAs among four tumors. **(A)** The hallmarks of different tumors. Blue represents COAD, red represents ESCA, green represents READ, and cyan represents STAD. **(B)** common differentially expressed mRNAs, **(C)** common differentially expressed miRNAs, **(D)** common differentially expressed lncRNAs. Blue represents COAD, red represents ESCA, green represents READ and yellow represents STAD.

In total, 3,999 mRNAs were identified as differentially expressed (DE) mRNAs in ESCA, including 1,776 up-regulated and 2,223 down-regulated mRNAs (**Supplementary Figure 2A** and **Supplementary Table 1**). For STAD, 6,520 mRNAs were identified as DE mRNAs, including 3,426 up-regulated and 3,094 down-regulated mRNAs (**Supplementary Figure 2B** and **Supplementary Table 1**). For COAD, 7,635 mRNAs were screened as DE mRNAs, including 4,203 up-regulated and 3,432 down-regulated mRNAs (**Supplementary Figure 2C** and **Supplementary Table 1**). And there were 7,184 DE mRNAs in READ, consisting of 3,780 up-regulated mRNAs and 3,404 down-regulated mRNAs (**Supplementary Figure 2D** and **Supplementary Table 1**). Finally, the number of common mRNAs was 1,700 among four tumors (**Figure 1B**).

Altogether, 187 miRNAs were considered as DE miRNAs in ESCA including 106 up-regulated and 81 down-regulated miRNAs (**Supplementary Figure 2E** and **Supplementary Table 2**). For STAD, 103 DE miRNAs were screened out, including 85 up-regulated and 18 down-regulated miRNAs (**Supplementary Figure 2F** and **Supplementary Table 2**). For COAD, 231 DE miRNAs were called out, including 133 up-regulated and 98 down-regulated miRNAs (**Supplement Figure 2G** and **Supplement Table 2**). Besides, there were 245 DE miRNAs in READ, including 174 up-regulated and 71 down-regulated miRNAs (**Supplementary Figure 2H** and **Supplementary Table 2**). In the end, there were 29 common DE miRNAs among the digestive tract cancers (**Figure 1C**).

We got 1,448 DE lncRNAs in ESCA, consisting of 744 up-regulated and 704 down-regulated lncRNAs (**Supplementary Figure 2I** and **Supplementary Table 3**). And for STAD, there were 4,422 DE lncRNAs, including 3,226 up-regulated and 1,196 down-regulated lncRNAs (**Supplementary Figure 2J** and **Supplementary Table 3**). And for COAD, there were 3,824 DE lncRNAs, including 2,584 up-regulated and 1,240 down-regulated lncRNAs (**Supplementary Figure 2K** and **Supplementary Table 3**). The number of DE lncRNAs in READ was 2,456, including 1,281 up-regulated and 1,175 down-regulated lncRNAs (**Supplementary Figure 2L** and **Supplementary Table 3**). Finally, the number of the common DE lncRNAs was 362 (**Figure 1D**).

Besides, as for the common up-regulated genes and down-regulated genes, the number of common up-regulated mRNAs was 769 and common down-regulated mRNAs was 808. About common up-regulated lncRNAs and down-regulated lncRNAs, the number was 216 and 135, respectively. Besides, there were 11 common up-regulated miRNAs and 12 common up-regulated miRNAs among the four cancers. And the common up-regulated genes and down-regulated genes were used to construct the ceRNA network.

### Gene Set Enrichment Analysis (GSEA) of Different Cancers

Aiming to explore the heterogeneity of digestive tract cancers, Gene set enrichment analysis (GSEA) was used to show the hallmarks of different cancers (18). The results of GSEA showed that 11 genes sets were enriched in ESCA such as G2/M checkpoint, E2F targets and KRAS signaling set. For STAD, the number of enriched gene sets was 14, including G2/M checkpoint, E2F targets and adipogenesis. And there were 11 terms enriched in COAD and 10 terms in READ. For instance, the gene sets were enriched in G2/M checkpoint, MYC targets and E2F targets in COAD and coagulation and MYC targets in READ (**Figure 1A** and **Supplementary Table 4**).

### GO and KEGG Pathways Enrichment Analysis of Common mRNAs

**Figure 2:**
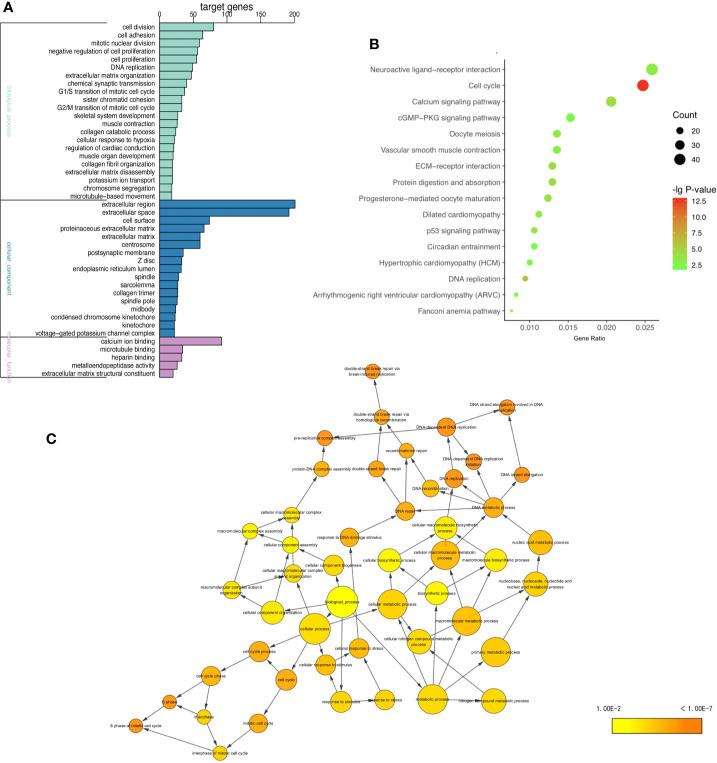
GO and KEGG enrichment analysis. **(A)** GO enrichment analysis including BP, CC, and MF, **(B)** KEGG pathway enrichment analysis, **(C)** the directed acycliic graph of GO terms. (P < 0.0001).

To gain insight into the biological functions of four tumors in common, GO and KEGG pathways analysis (11, 12) were performed on 1,700 shared DE mRNAs. GO terms were enriched in biological processes (BP) “DNA replication”, “cell division”, “G1/S transition of mitotic cell cycle”, and “mitotic nuclear division” of biological processes (**Figure 2A** and **Supplementary Table 5**). The KEGG pathways analysis showed that the common DE mRNAs were significantly enriched in “Cell cycle”, “DNA replication”, “ECM-receptor interaction”, and “Calcium signaling pathway” (**Figure 2B** and **Supplementary Table 6**). And Cytoscape BiNGo plug-in was utilized to visualize the major GO terms, which was shown in **Figure 2C** (13). Besides, to show the overall enrichment, we reset p-value <0.001 in **Supplementary Figure 3**.

### The Construction of ceRNA Network

**Figure 3:**
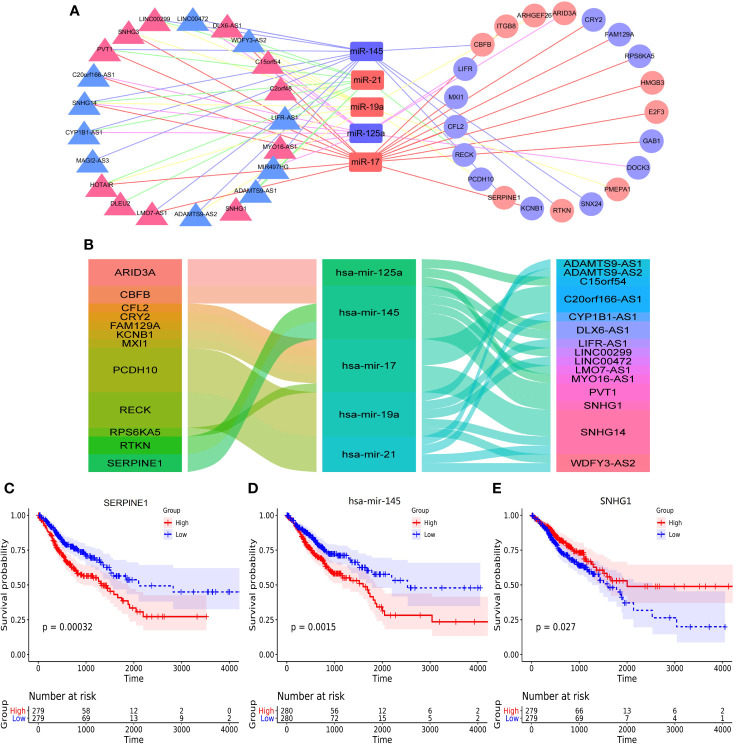
The construction of ceRNA network and overall survival analysis. **(A)** the construction of ceRNA network by cytoscape, **(B)** the alluvial diagram of regulatory network of ceRNA, **(C)** the overall survival of SERPINE1, **(D)** the overall survival analysis of hsa-mir-145, **(E)** the overall survival analysis of SNHG1.

The Competing endogenous RNAs (ceRNAs) regulate RNA transcripts by competitively binding with shared miRNAs (15). According to recent investigations, ceRNAs play important parts in cancer initiation and progression and have cancer-specificity, supporting ceRNAs serve as diagnostic biomarkers or therapeutic targets (19). In the present study, the ceRNA network showed that there were 5 core miRNA nodes, 21 lncRNA nodes, 21 mRNAs nodes, and 66 edges (**Figure 3A**). We selected the negative interactions from the ceRNA network to construct regulatory axes, including 16 lncRNAs, 5 miRNAs, and 12 mRNAs (**Figure 3B**). For the lncRNA, the small nucleolar RNA host gene 14 (SNHG14), which had the highest connectivity and co-expression relationships with mRNAs of the ceRNA network, the ImmunLnc database showed that SNHG14 took part in immune pathways such as antigen processing and presentation in four tumors (**Supplementary Figures 4C–J**).

The correlations between ceRNAs and the survival outcomes of patients were analyzed to validate the prognostic value. The results showed serpin peptidase inhibitor (SERPINE1), hsa-mir-145 and small nucleolar RNA host gene 1 (SNHG1) were significantly correlated with overall survival (**Figures 3C–E**). Besides, they formed a potential regulatory axis SERPINE1/hsa-mir-145/SNHG1 based on sponge effect in ceRNA network. And the correlations between the above three genes and overall survival were shown in **Supplementary Figure 5**.

### The Weighted Gene Co-Expression Network Analysis (WGCNA)

**Figure 4:**
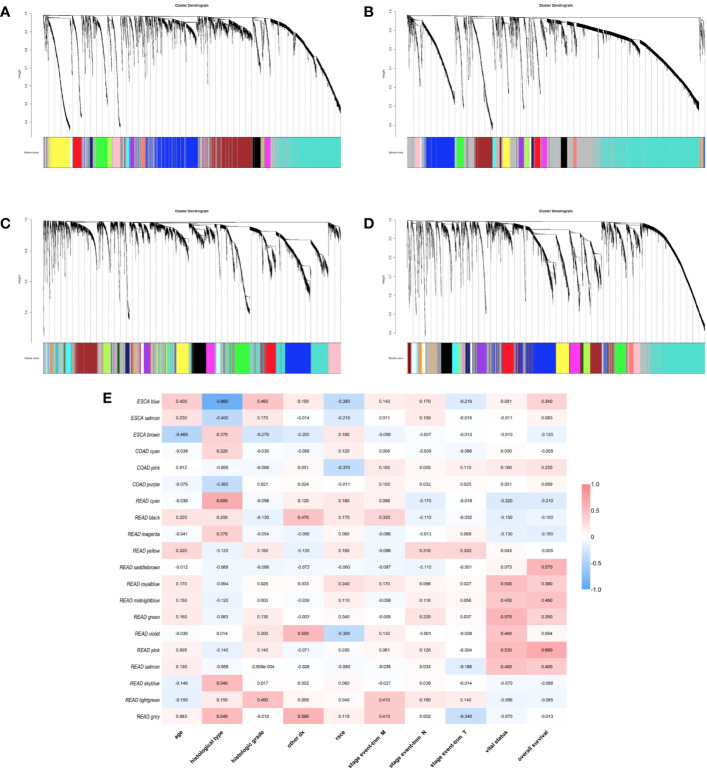
Identification of weighted gene co-expression network modules. Different colors represent different gene modules **(A)** ESCA, **(B)** STAD, **(C)** COAD, and **(D)** READ. **(E)** the heatmap of correlation between modules and clinical traits.

We used gene co-expression network analysis to screen gene modules which were closely related to clinical traits. For ESCA, transcriptome were divided into 18 modules and the blue, brown, and salmon modules were related to clinical traits (**Figures 4A, E**). For STAD, genes were divided into 22 modules and there was no module screened associated to clinical traits (**Figure 4B**). For COAD, genes were divided into 34 modules and the cyan, pink, and purple modules were screened as the modules which were related to clinical traits (**Figures 4C, E**). For READ, the genes were divided into 33 modules and 14 of them were screened correlated to clinical traits (**Figures 4D, E**). Thus, there were 20 modules related to clinical traits in total. The relationships between other modules and clinical traits were shown in **Supplementary Figure 6**.

**Figure 5:**
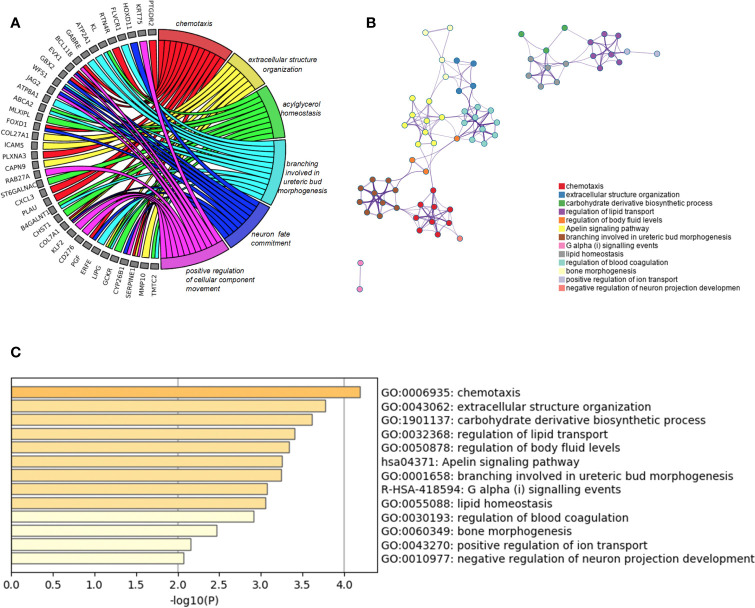
GO enrichment analysis. **(A)** the relation between common genes and GO terms of ESCA and READ, **(B)** Network of enriched terms: Colored by cluster ID, **(C)** the P-value of GO terms of ESCA and READ.

**Figure 6:**
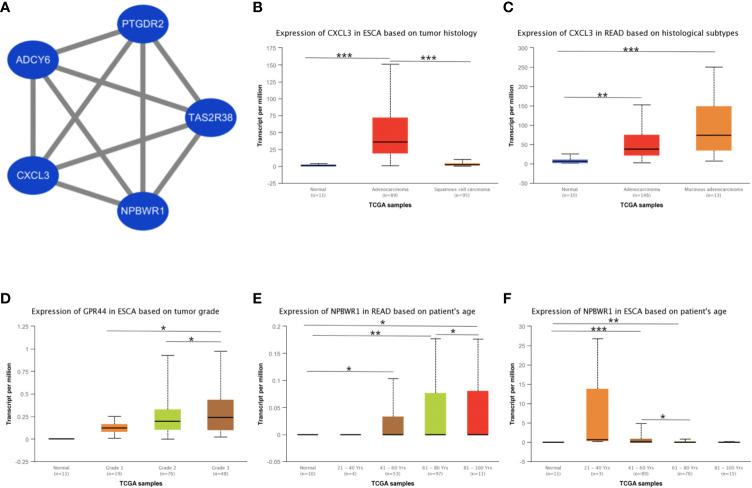
The identification of hub genes and the correlation between hub genes expression and clinical traits. **(A)** the interaction network of hub genes, **(B)** the correlation between CXCL3 expression and tumor histological types in ESCA, **(C)** the correlation between CXCL3 expression and tumor histological types in READ, **(D)** the correlation between GPR44 (PTGDR2) expression and tumor grades in ESCA, **(E)** the correlation between NPBWR1 expression and ages in READ, **(F)** the correlation between NPBWR1 expression and ages in ESCA. *p < 0.05, **p < 0.01, ***p < 0.001.

Besides, we took the intersection of modules related to the same clinical traits among different cancers. As a result, ESCA and READ had three common genes in neoplasm histologic grade and three common genes in the trait of age. And there were 30 common genes in the trait of overall survival between ESCA and READ. For the trait of race, we found 10 common genes between ESCA and COAD. Last but not the least, about the trait of histological types, there were 4 genes shared by ESCA, COAD and READ, 31 common genes between READ and ESCA, 32 common genes between COAD and ESCA and 26 common genes between COAD and READ. Thus, in total, the number of common genes between ESCA and READ was 67. Then between COAD and ESCA the number was 42 and between COAD and READ being 26. For the common genes between different tumors, GO and KEGG pathways enrichment analysis were performed to show the functions of these genes (**Supplementary Figure 7**). Considering that the number of common genes between READ and ESCA was the highest, these 67 common genes were performed further analysis. The results showed that they were significantly enriched in extracellular structure organization, chemotaxis, and positive regulation of cellular component movement (**Figures 5A–C**). And it screened out 5 hub genes of the 67 genes by protein-protein interaction (PPI) by cytoscape Mcode plug-in (**Figure 6A**) (17). Among them, the UALCAN database showed that CXCL3 was related to the trait of histological types in ESCA and READ, GPR44 (PTGDR2) was associated with tumor grade in ESCA and NPBWR1 was related to patients’ age in ESCA and READ (**Figures 6B–F**). Furthermore, Disease Meth version 2.0 analysis showed that the mean methylation levels of NPBWR1 was higher in tumor tissue than normal tissue both in ESCA and READ (P < 0.01) (**Supplementary Figures 8A, B**).

In order to verify above genes representing the common features in alimentary tract malignancies, the exploration of database TIMER2.0 (http://timer.cistrome.org/) showed that SERPINE1, SNHG1, ADCY6, CXCL3, ADCY6, CXCL3, NPBWR1, TAS2R38, and PTGDR2 were mainly differentially expressed in alimentary tract malignancies (**Supplementary Figure 9**).

## Discussion

The morbidity of alimentary tract malignancies has declined though, it is still the leading cause of death in malignant tumors worldwide nowadays (2). Thus, it remains an essential task to explore the carcinogenesis and mechanisms in tumor growth. There are many investigations using RNA-seq to discover novel biomarkers and therapeutic targets for the digestive tract cancers, such as miR-378, miR-199a, and miR-92a which are closely related to survival rate of colon cancer (20). However, lots of investigations put emphasis on a certain type of cancer but relatively little concrete on their homogeneity. According to recent researches, upper gastrointestinal (GI) tumors with chromosomal instability are characterized in fragmented genomes and lower GI tumors are enriched in mutations in SOX9 and PCBP1, which reveal the heterogeneity between upper GI tumors and lower GI tumors (21).

In this study, heterogeneity is analyzed among four tumors from the point of tumor hallmarks (22). And we mined 1,700 common DE mRNAs, 336 DE lncRNAs, and 29 DE miRNAs, mainly involved in functional terms where pathways and gene clusters are widely reported in recent investigations and play important roles in the development of digestive tract cancers (23–27).

By the construction of ceRNA network, interactions among various types of RNA molecules reveal the frank regulation inside the whole transcriptome. Herein, TCGA malignancies of alimentary tract share the core ceRNA network with nodes of 5 miRNAs, 21 lncRNAs, and 21 mRNAs. The SNHG14, one of lncRNAs, had the highest connectivity, with all 5 miRNAs in the network. Recently, it is reported that high expression of SNHG14 in tumor tissue promotes cancer cell invasion and metastasis by targeting hsa-mir-145 in gastric cancer and hsa-mir-944 in colorectal cancer (28, 29). On the contrary, there are some investigations showing that up-regulation of SNHG14 suppresses cell proliferation and metastasis of colorectal cancer by targeting hsa-mir-92b and hsa-mir-186 (30, 31). Intriguingly, the data of GEPIA2 showed that SNHG14 is a down–regulated lncRNA in gastrointestinal cancers in comparison to normal tissue and its expression is positively correlated with stage development (**Supplementary Figures 4A, B**). These contradictory results are probably caused by that they set different normal cell lines as control groups. For instance, the expression of SNHG14 in FHC cell lines is higher than other cancer cells but is lower in NCM460 cell lines (28, 30–33). Thus, the function of SNHG14 is complicated and might serve as both positive and negative roles in cancer initiation and progression corresponding to bio-context. Moreover, SNHG14 is associated with immunity and could be a promising target for the immunotherapy through PD-L1/PD-1 blockade in cancers (34). Our exploration based on ImmunLnc database supports this story that SNHG14 is associated with antigen processing and presentation (**Supplementary Figure 4J**).

As for prognostic values, our research illustrates that SERPINE1, hsa-mir-145, and SNHG1 are closely related to survival rate in the common ceRNA network of four digestive tract tumors. Hsa-mir-145 is an important microRNA widely regarded as a tumor suppressor, of which expression is down-regulated in various cancers (35). Our research shows that hsa-mir-145 is related to the overall survival of gastrointestinal cancers, consistent with latest finding in COAD, ESCA, and STAD (36–38). Besides, it is reported that SERPINE1 is significantly associated with poor outcomes in gastric cancer and a potential biomarker for tumor aggressiveness in colorectal cancer (39, 40). Moreover, as a member of small nucleolar RNA host lncRNA family, SNHG1 acts as a useful tumor biomarker for cancer diagnosis, prognosis, and treatment (41). Three molecules above form a ceRNA regulatory axis, in which our research prompts that SNHG1 possibly performed as sponge to provoke the effect of hsa-mir-145 on the up-regulation of SERPINE1, enhancing tumor cell migration and invasion (42). Meanwhile, the SERPINE1/hsa-mir-145/SNHG1 axis is associated with overall survival and could serve as a critical target for therapy. Besides, to explore therapeutic value of the key nodes in other cancers, we found that SERPINE1/hsa-mir-145/SNHG1 axis mainly expressed in alimentary tract malignancies but not other cancers.

WGCNA is an R package for weighted correlation network analysis and is widely used in bioinformatics analysis. In order to explore the relationships between genes and clinical features, the R WGCNA package is used to construct Co-expression networks. WGCNAs yield 20 modules related to clinical traits and reveal common features shared between ESCA and READ. For instance, ESCA and READ have 67 common genes in the clinical traits of age, histological types, neoplasm histologic grade and overall survival. About the GO and KEGG pathways enrichment, the 67 common genes are related to chemotaxis, extracellular structure organization, acylglycerol homeostasis and regulation of lipid transport. Considering the similarities of ESCA and READ, esophagus and rectum are the beginning and end parts of the alimentary tract, both consisting of stratified squamous epithelium and simple columnar epithelium and functioning as the cargo of food or residue on the border between inner digestive system and outside. Considering their similar biofunction and bi-category tissue constitution, ESCA and READ should share the common epithelial carcinogenesis.

To explore the relationships of common genes between ESCA and READ, we screen the hub genes including, NPBWR1, TAS2R38, CXCL3, ADCY6, and PTGDR2, which are closely related to clinical traits and mainly express in alimentary tract malignancies. Neuropeptide B/W receptor-1 (NPBWR1/GPR7), a G protein-coupled receptor, implicates in the biological process of energy homeostasis, neuroendocrine function and modulating inflammatory pain (43, 44). And methylation of NPBWR1 is significantly associated with prostate cancer prognosis (45). Similar with prostate, our research proves NPBWR1 expression in ESCA and READ is significantly associated with its methylation. From the data of MethSurv, we find that with age increasing, the methylation level is increasing, contrary to the expression of NPBWR1 (**Supplementary Figure 8D**). Thus, it can be deduced that age influences the expression of NPBWR1 by affecting the methylation level in ESCA. Similar with NPBWR1, PTGDR2, also named CRTH2 and GPR44, is another G protein-coupled receptor and the receptor of PGD2, preferentially expressing in CD4+ effector T helper 2 cells (46). Previous studies show that PTGDR2 inhibits tumor growth and tumorigenesis in gastric cancer and restricts angiogenesis in colon cancer (47, 48). Our research shows that PTGDR2 is associated with the tumors’ grades in ESCA and its expression is higher with tumor grades increasing.

For the other hub genes, ADCY6 encodes a member of the adenylate cyclase protein family, and it is required for the synthesis of cAMP (49). In our research, the ADCY6 is associated with the clinical traits of overall survival in READ and ESCA. For the hub gene TAS2R38, encoding bitterness taste receptor, influences the progression of adenomatous polyps by dietary vitamin C and folic acid (50). Other studies show that genetic variation in TAS2R38 is thought to be associated with gastrointestinal risks by modifying dietary intake (51, 52). In our research, this gene is associated with the trait of overall survival and the mean methylation levels of TAS2R38 was lower in READ (**Supplementary Figure 8C**).

CXCL3 is also known as CINC-2 alpha (Cytokine-induced neutrophil Chemoattractant) and is a number of signaling pathways such as ERK1/2 MAPK, by activating CXCR2 receptor (53–55). There are investigations showing that CXCL3 is associated with vascular invasion and tumor capsule formation in HCC and is targeted by IRF2 to suppress MDSC migration and infiltration in colorectal cancer (54, 56). In our research, we find that the CXCL3 is associated with tumor histology types and is in high expression in adenocarcinoma, especially in Mucinous adenocarcinoma. Besides, these five hub genes all take part in the G alpha (i) signaling pathway. NPBWR1 and PTGDR2 are both G protein-coupled receptors and TAS2R38 encodes a seven-transmembrane G protein-coupled receptor associated with taste glucosinolates (44, 57, 58). ADCY6 catalyzes the formation of the signaling molecule cAMP downstream of G protein-coupled receptors (49, 59). And CXCL3 encodes a secreted growth factor that signals through the G-protein coupled receptor, CXC receptor 2 (60, 61). Thus, these genes have co-expression relationships in G alpha (i) signaling pathway and reason the similarity in ESCA and READ.

In conclusion, by constructing ceRNA network and using WGCNA analysis, we find a potential regulatory axis and several significant gene modules in alimentary tract cancers. For the clinical treatment of these malignancies, the axis SERPINE1/hsa-mir-145/SNHG1 could serve as prognostic biomarkers and potential therapeutic targets. The hub gene ADCY6, CXCL3, NPBWR1, TAS2R38, and PTGDR2 highly influence the clinical traits of age, histology types, neoplasm histologic grade, and overall survival in ESCA and READ, leading to their similarity in these clinical traits. Further study is guaranteed for sure, aiming to identify the therapeutic value of SERPINE1/hsa-mir-145/SNHG1 axis and fully reveal the hub genes contribution to the clinical outcomes.

## Data Availability Statement

The datasets presented in this study can be found in online repositories. The names of the repository/repositories and accession number(s) can be found in the article/**Supplementary Material**.

## Author Contributions

KY and D-BJ developed the original idea. KY, D-BJ, and L-HZ designed the whole study. Y-CL, J-QS, Z-XZ, and J-YZ ran the most of data processing. Y-CL, H-KZ, and Y-CF did data visualization. Z-HL and Z-CL helped to access the public data. S-YY, X-YZ, YL, and Y-JS reviewed the manuscript and raised suggestions. Y-CL and D-BJ drafted the manuscript. D-BJ and J-QS revised the writings. All authors contributed to the article and approved the submitted version.

## Funding

This study was supported by the National Natural Science Foundation of China (No. 81772763 and No. 82073154) and Key Research and Development Program of Shaanxi Province (No. 2020SF-200).

## Conflict of Interest

The authors declare that the research was conducted in the absence of any commercial or financial relationships that could be construed as a potential conflict of interest.
