# Supplementary material for: Transcriptome Based System Biology Exploration Reveals Homogeneous Tumorigenicity of Alimentary Tract Malignancy
Source: Front Oncol. 2021 Jan 22;10:580276. doi: 10.3389/fonc.2020.580276 (PMC7862768; doi:10.3389/fonc.2020.580276)
Supplement: Supplementary file 1 [file Image_1.pdf]

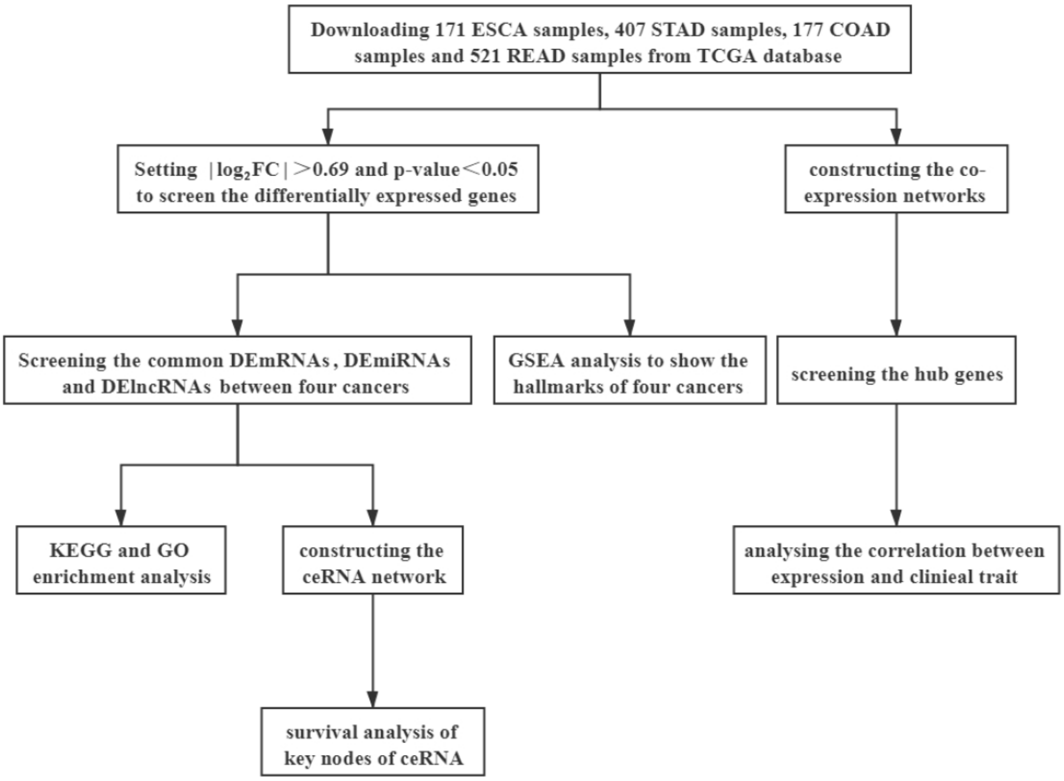

Supplementary figure 1. Flow chart of data downloading, processing, analysis, and validation.

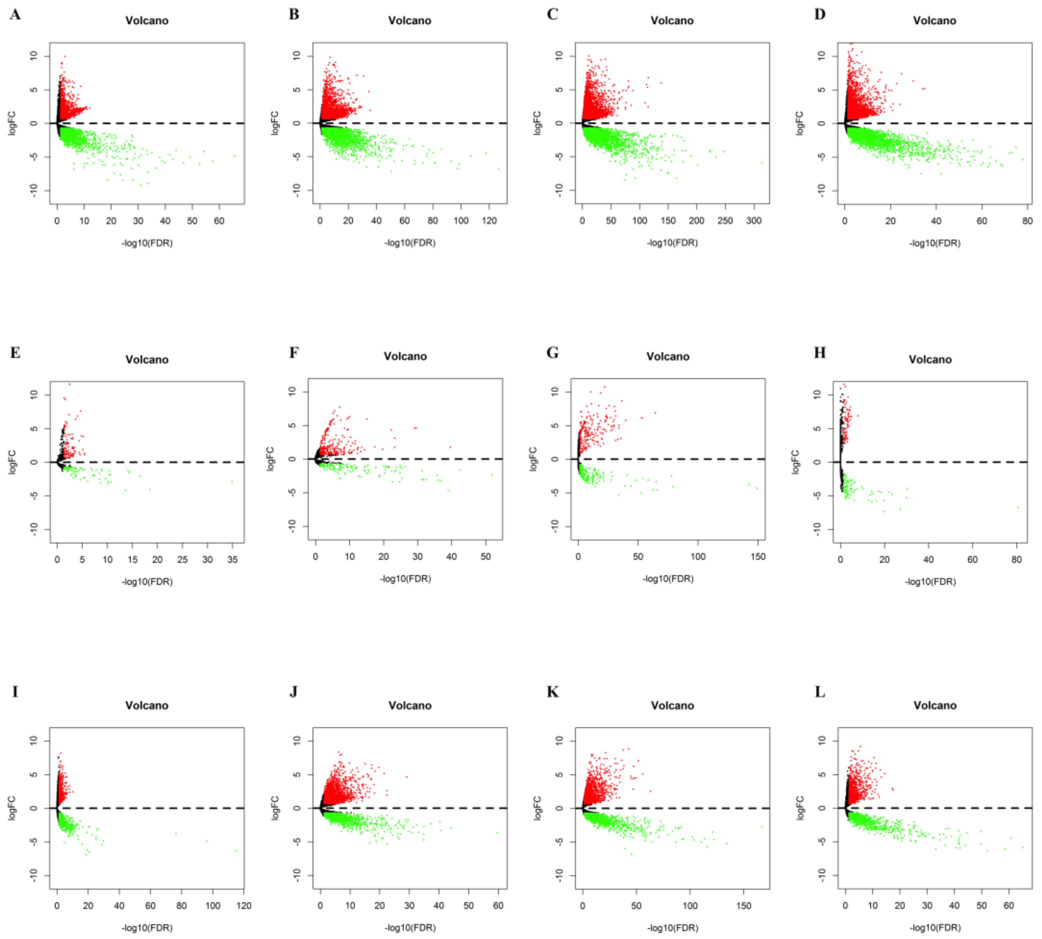

Supplementary figure 2. The volcano diagram of mRNA, miRNA and lncRNA for four tumors. Red represents up-regulated genes and green represents down-regulated genes. (A-D) differentially expressed mRNAs of ESCA, STAD, COAD and READ, (E-H) differentially expressed miRNAs of ESCA, STAD, COAD and READ, (I-L) differentially expressed lncRNAs of ESCA, STAD, COAD and READ.

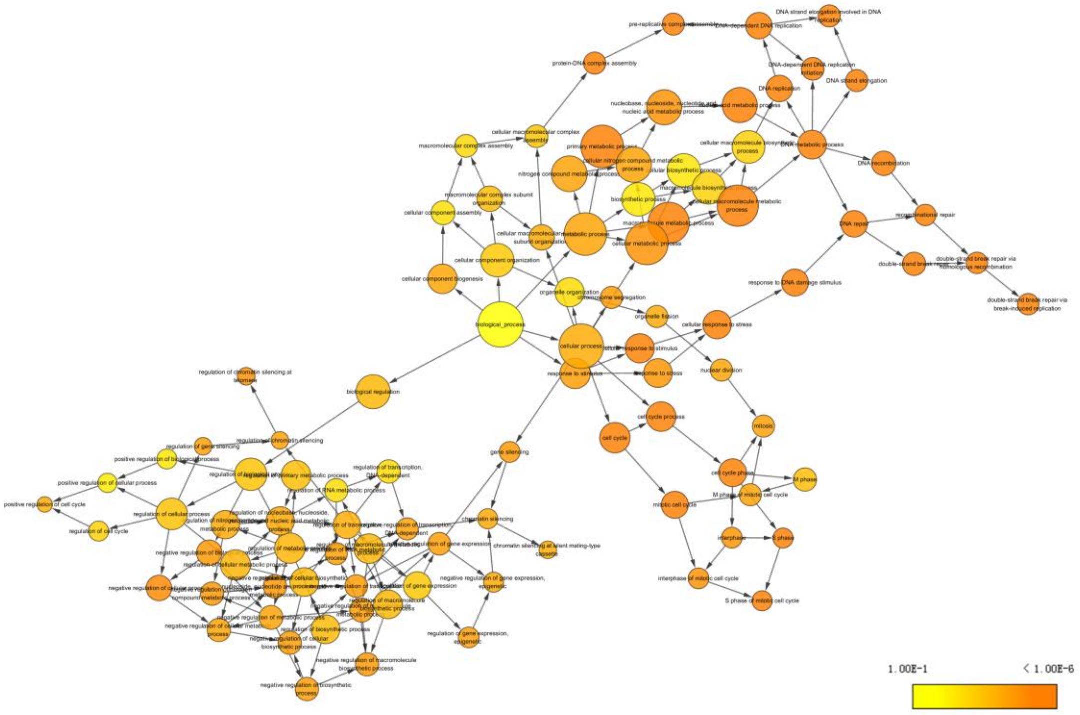

Supplementary figure 3. The directed acyclic graph of GO terms (p<0.001).

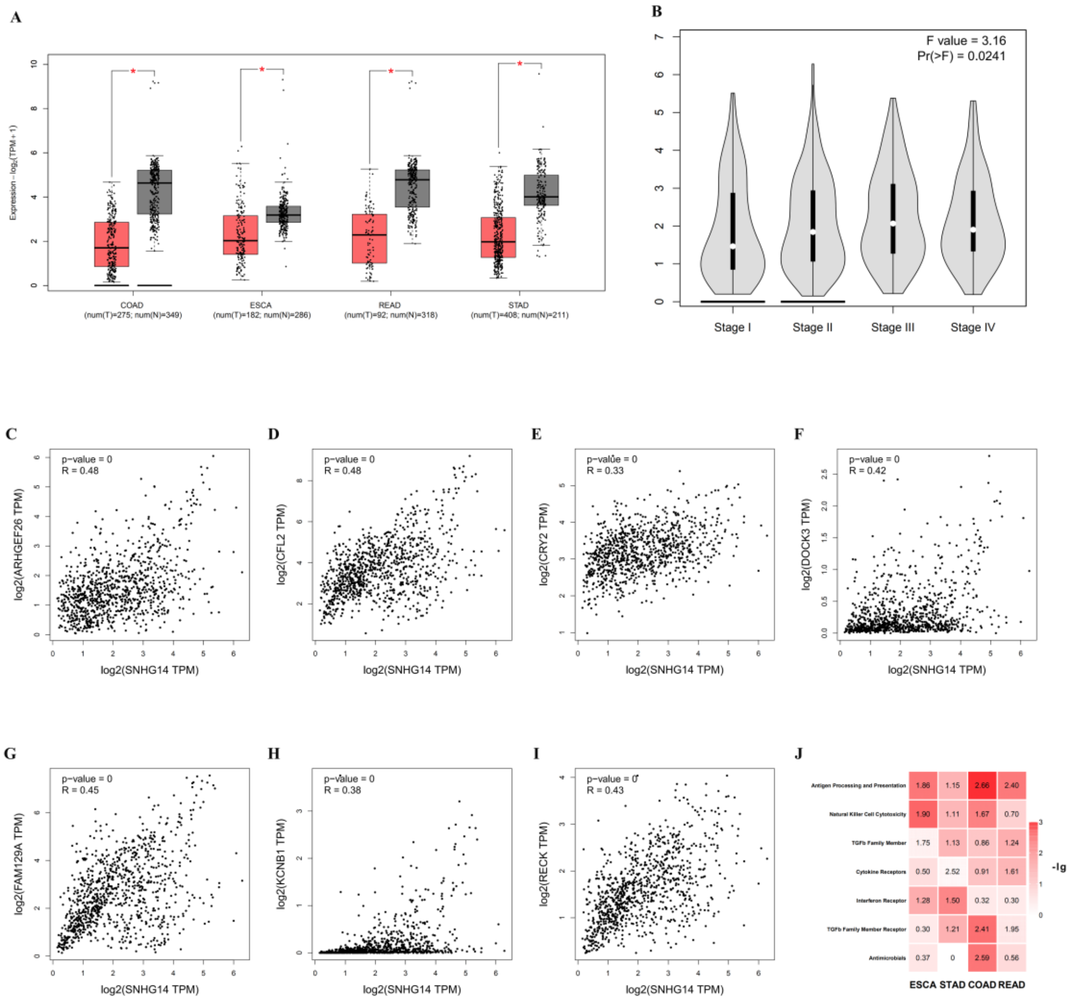

Supplementary figure 4. The gene expression, correlation genes analysis and immune pathways analysis of SNHG14. (A) the expression of SNHG14 in four tumors and normal tissue, (B) the expression of SNHG14 in different stages, (C-I) the correlation analysis between SNHG14 and mRNAs in ceRNA network, (J) the immune pathways analysis of SNHG14.

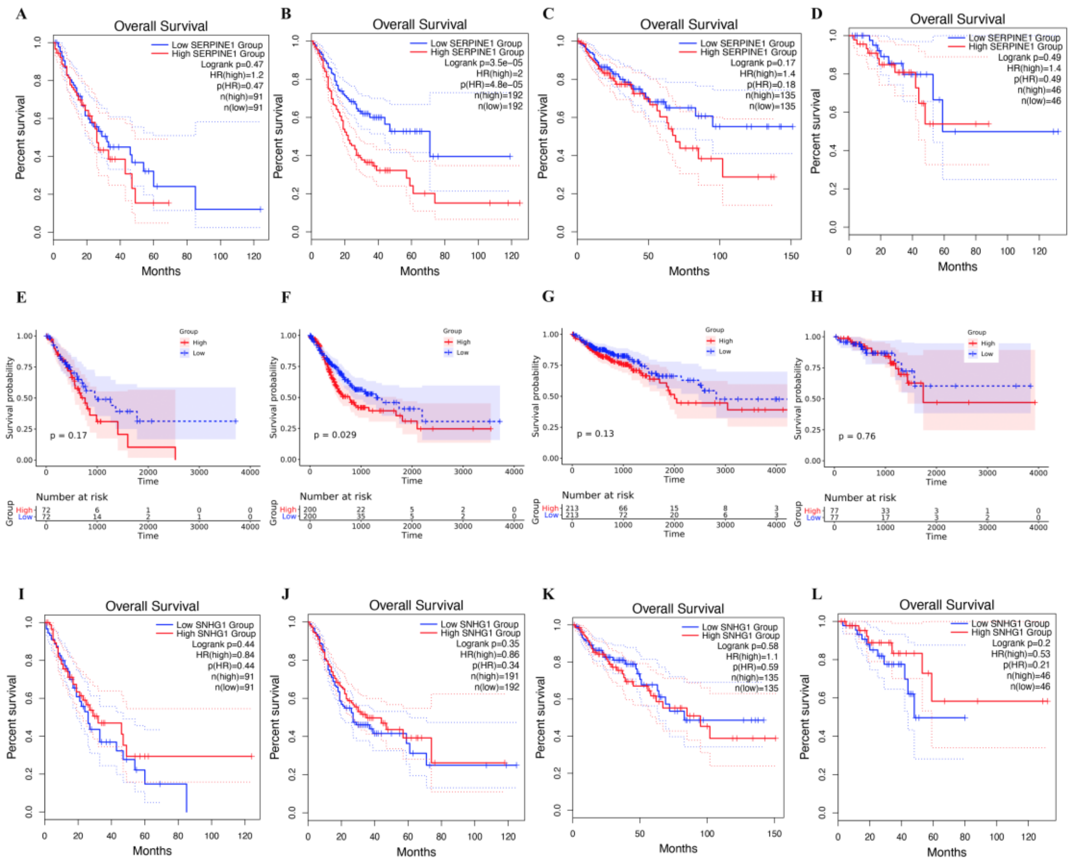

Supplementary figure 5. The overall survival analysis of SERPINE1, hsa-mir-145 and SNHG1 in four tumors. (A-D) the overall survival of SERPINE1 in ESCA, STAD, COAD and READ, (E-H) the overall survival analysis of hsa-mir-145 in ESCA, STAD, COAD and READ, (I-L) the overall survival analysis of SNHG1 in ESCA, STAD, COAD and READ.

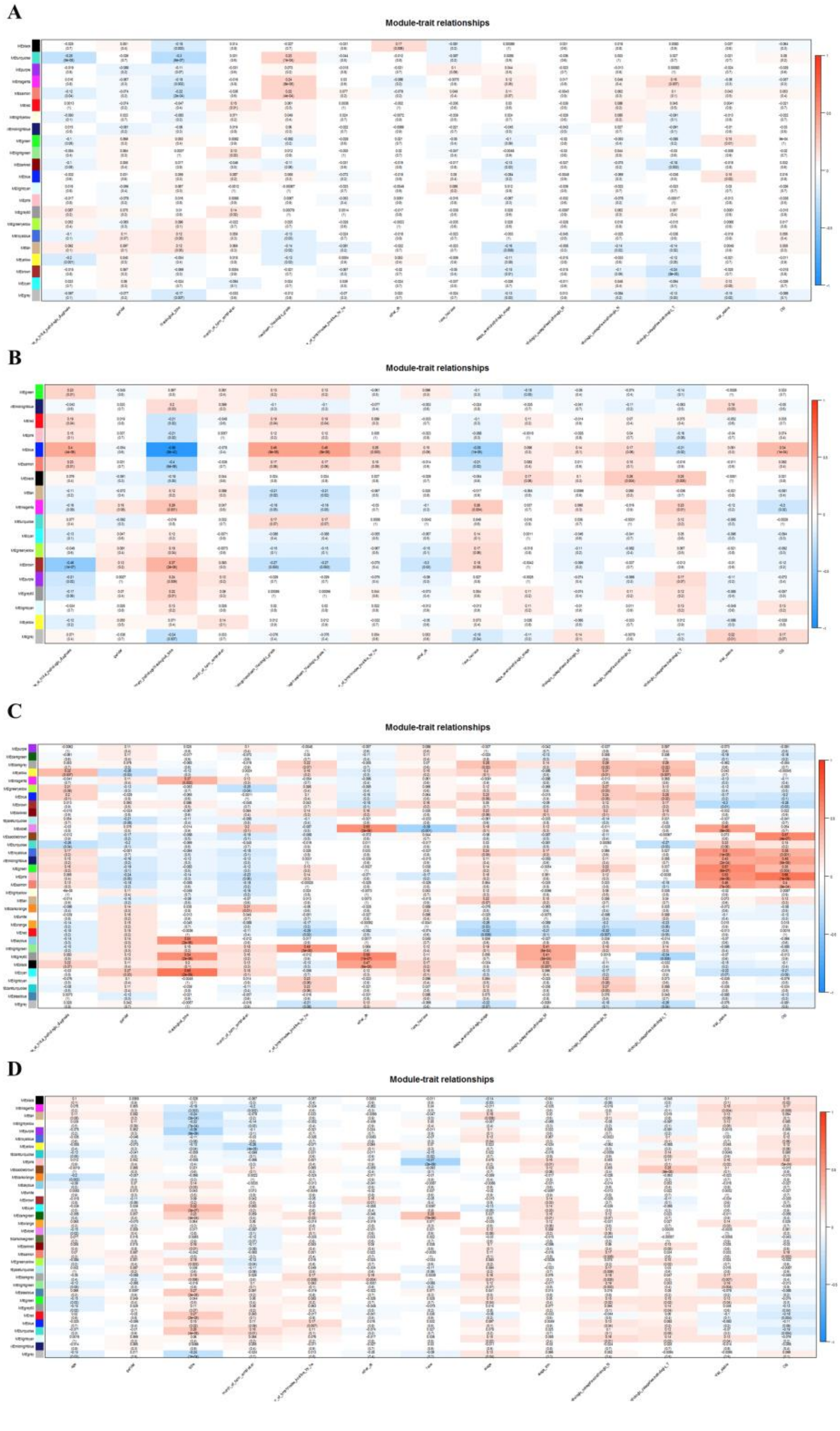

Supplementary figure 6. the heatmaps of correlation between co-expressing genes modules and clinical traits in four tumors. (A) ESCA, (B) STAD, (C) COAD and (D) READ.

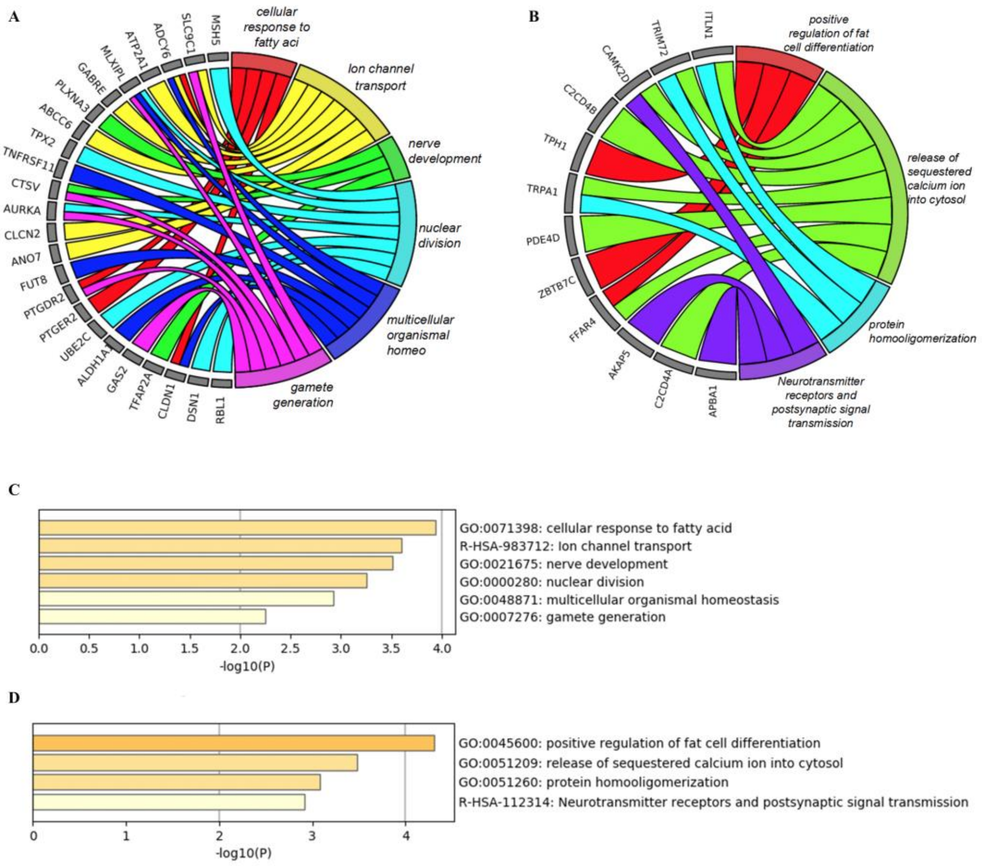

Supplementary figure 7. GO enrichment analysis of shared co-expression genes between ESCA and COAD, COAD and READ. (A) the relation between common genes and GO terms of ESCA and COAD, (B) the relation between common genes and GO terms of COAD and READ, (C) the P-value of GO terms of ESCA and COAD, (D) the P-value of GO terms of COAD and READ.

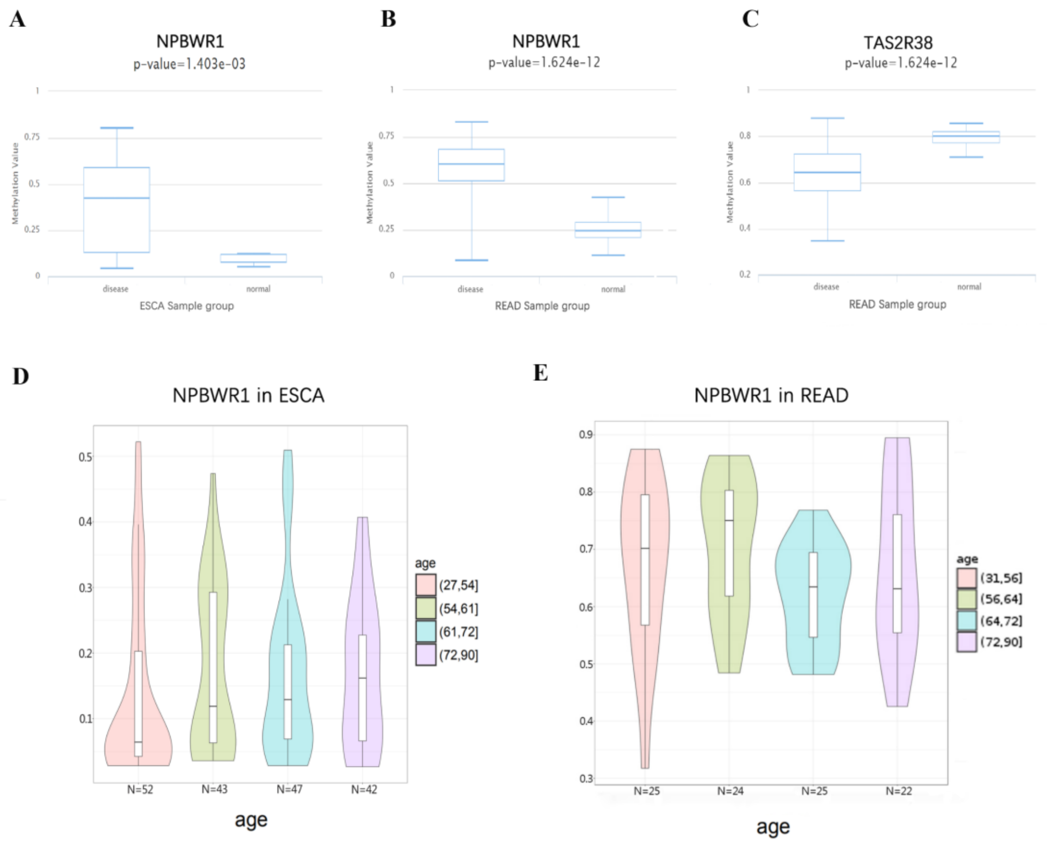

Supplementary figure 8. Methylation analyses of NPBWR1 and TAS2R38 in ESCA and READ. The methylation levels of (A) NPBWR1 in ESCA and normal tissues, (B) NPBWR1 in READ and normal tissues, and (C) TAS2R38 in READ and normal tissues, (D) The methylation levels of NPBWR1 of different ages groups in ESCA, (E) The methylation levels of NPBWR1 of different ages groups in READ.

A

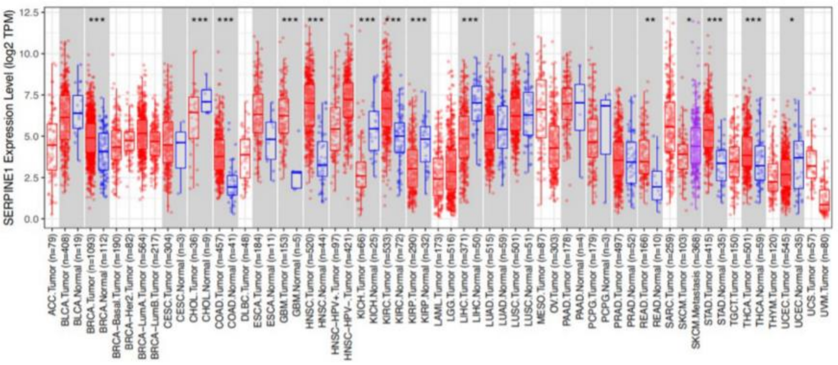
SERPINE1 Expression Level (log2 TPM)

B

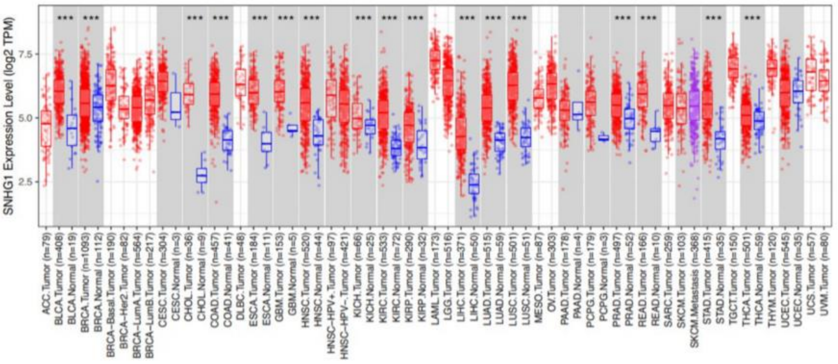
SNHG1 Expression Level (log2 TPM)

C

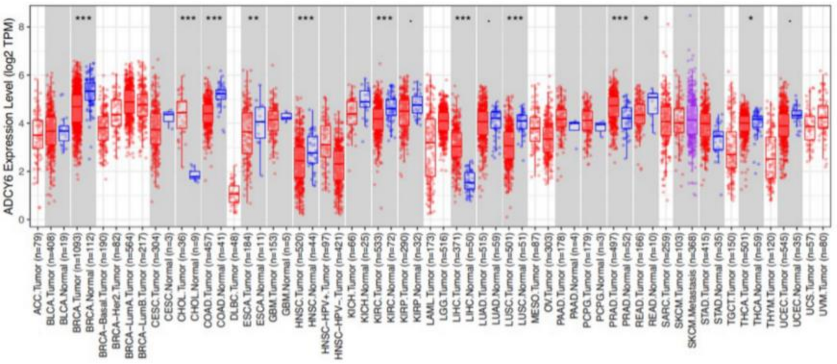
ADCY6 Expression Level (log2 TPM)

D

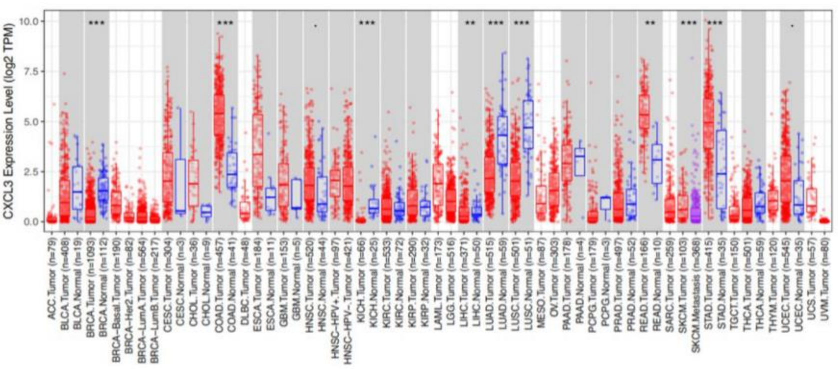
CXCL3 Expression Level (log2 TPM)

E

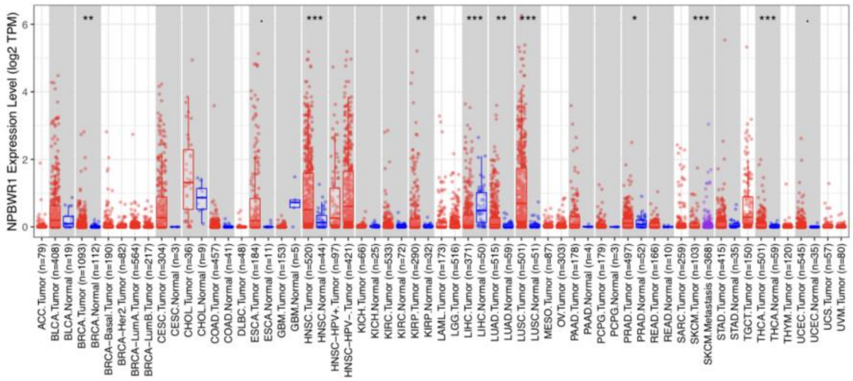

F

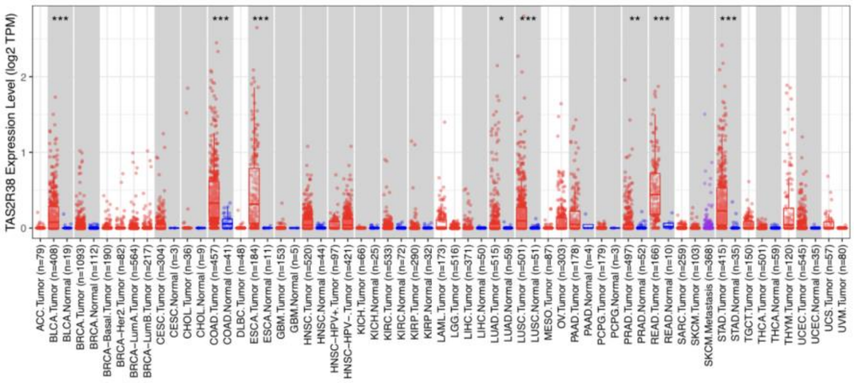

G

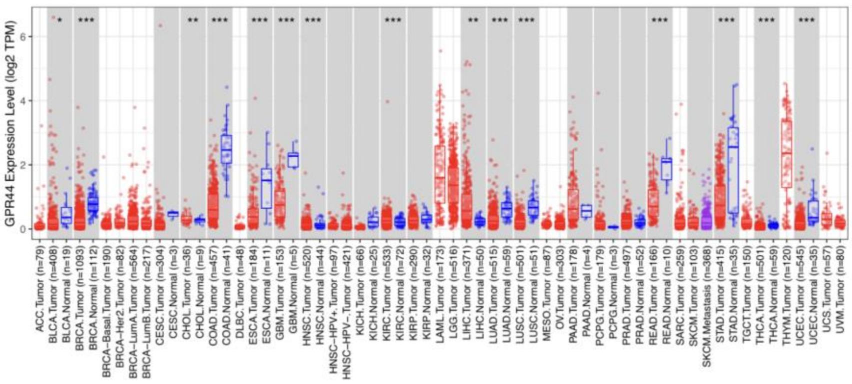

Supplementary figure 9. The gene expression levels between tumor and normal tissue among all The Cancer Genome Atlas datasets were analyzed by the Tumor Immune Estimation Resource (A) SERPINE1 (B) SNHG1 (C) ADCY6 (D) CXCL3 (E) NPBWR1 (F)TAS2R38 and (G) PTGDR2. (*P < 0.05, **P < 0.01, ***P < 0.001).
